# Bibliometric analysis of pharmacist’s research on antimicrobial stewardship in Japan: an interrupted time series analysis on the implementation of the certification system for infection control pharmacists

**DOI:** 10.1186/s40780-021-00223-w

**Published:** 2021-11-01

**Authors:** Masayuki Maeda, Takefumi Miyake, Ryo Inose, Satoru Ueda, Ken-ichi Matsugi, Yuichi Muraki, Takashi Kitahara

**Affiliations:** 1 Division of Infection Control Sciences, Department of Clinical Pharmacy, School of Pharmacy, Showa University, 1-4-5 Hatanodai, Shinagawa-ku, Tokyo, Japan; 2 Fourth Subcommittee, Committee on Academic, The Japanese Society of Hospital Pharmacists, Tokyo, Japan; 3 Department of Pharmacy, Kyoto Social Welfare Foundation Nishijin Hospital, Kyoto, Japan; 4 Department of Clinical Pharmacoepidemiology, Kyoto Pharmaceutical University, Kyoto, Japan; 5 Department of Pharmacy, Tazuke Kofukai Medical Research Institute, Kitano Hospital, Osaka, Japan; 6 Pharmaceutical Department, Kansai Electric Power Hospital, Osaka, Japan; 7 Clinical Pharmacology, Graduate School of Medicine, Yamaguchi University, Yamaguchi, Japan

**Keywords:** Antimicrobial stewardship, Japan, Bibliometric analysis, Pharmacist

## Abstract

**Background:**

Pharmacist plays an integral role in promoting antimicrobial stewardship (AS) strategies by committing to the evidence-based activities in this field. The present study aims to document trends in actual achievements through bibliometric analysis and identify the future direction of pharmacists with expertise in AS by describing the characteristics of articles on AS written by Japanese pharmacists.

**Methods:**

The study searched for articles written in Japanese and English on Ichushi-Web and MEDLINE, respectively, until December 2020 for published articles relevant to AS. The articles were classified into the seven groups according to content. Interrupted time series analysis (ITSA) was performed to identify the effect of the certification system for infection control pharmacy specialists (ICPSs) on the number of articles in Japanese.

**Results:**

The study retrieved 476 and 145 titles from Ichushi-Web and MEDLINE, respectively, out of which 383 and 123 articles written in Japanese and English, respectively, were considered relevant to AS. A continued publication was found for Japanese articles written by pharmacists assigned to large-sized hospitals since 1998, whereas few articles in English were published until 2017. The most frequent content of articles in both languages was intervention (56.7 and 59.0%, respectively). ITSA indicated that the number of publication slightly increased before [*β*1 = 1.33, 95% confidence interval (CI): − 0.62–3.28; *P* = 0.169] the implementation of the system. Moreover, the level (*β*2 = 11.41, 95%CI: − 0.23–23.05; *P* = 0.054) increased after the implementation of the system, whereas the slope decreased (*β*3 = − 2.07, 95%CI: − 4.16–0.03; *P* = 0.053). However, the changes were not statistically significant.

**Conclusion:**

The study identified the contribution of pharmacists by documenting trends in AS practice and by conducting bibliometric analysis. The implementation of the ICPS certification system positively influenced the trend of publications. Therefore, the study recommends that policymakers and stakeholders should promote and support the evidence-based activities for AS for pharmacists in small- to medium-sized hospitals.

**Supplementary Information:**

The online version contains supplementary material available at 10.1186/s40780-021-00223-w.

## Background

Antimicrobial stewardship (AS) is important for public health and aims to conserve the effectiveness of antimicrobials and to promote appropriate antibiotic use. Pharmacists play an integral role in promoting AS strategies by committing to the evidence-based activities in this field [1, 2].

Pharmacists should be acknowledged as health care professionals specialising in pharmacotherapy outcomes and management. Several countries, such as the United States and Japan, have implemented the board certification system for pharmacy specialties [3, 4]. Specifically, the Japanese Society of Hospital Pharmacists launched a certification system for infection control pharmacy specialists (ICPSs) on fiscal year 2005 [4, 5]. As of 2005, the requirements for certification were broad knowledge and skills of infection control and AS, which include at least two publications of original articles. Although, scientific research publication by pharmacists are speculated to increase, specific data regarding the trend of publication relevant to AS remain lacking.

In recent year, interest in AS among Japanese pharmacists became widespread due to guidelines issued by a joint committee consisting of eight societies, including the Japanese Society of Chemotherapy (2017) [6], and the enforcement of additional reimbursement for AS (2018) [7]. Several nationwide surveys revealed that these directions promoted the commitment of pharmacists to AS [8–10]. However, these studies also reported that a shortage exists for pharmacists with expertise in AS among the majority of Japanese hospitals [8, 10]. To set the direction for efforts for AS in the future, evidence-based policy making, such as a certification system, is required to develop pharmacists with expertise in AS.

Thus, the present study aims to reveal the trends in actual achievements in this field using bibliometric analysis and to identify the future direction of pharmacists with expertise in AS by describing the characteristics of articles on AS written by Japanese pharmacists.

## Materials and methods

### Database and literature search

The study searched for articles related to AS, which written in English and Japanese on PubMed/MEDLINE and Ichushi-Web, respectively, and which were published until December 2020. Additional file 1 provides the search terms used.

Two authors (M. M. and Y. M.) exported all potentially relevant studies into Excel Microsoft®. All authors, except for M.M. initially vetted and categorised all titles and abstracts of potentially relevant studies based on the study criteria and definition. The authors retrieved full texts as required to confirm whether the studies matched the criteria or definition. An independent reviewer (M. M.) re-reviewed all selected articles.

### Inclusion and exclusion criteria

Studies that met the inclusion criteria consisted of any type of article relevant to AS in Japan. Studies were excluded if they are basic research, articles relevant to infection prevention and control, reports on facilities outside Japan, and AS targeting antiviral agents and disinfectants.

### Definition of literature category

The study categorised the selected studies into the following groups: (1) intervention relevant to AS, (2) therapeutic drug monitoring of antibiotics, (3) surveillance or evaluation relevant to AS without intervention, (4) evaluation of efficacy or adverse event of antibiotics, (5) case report; (6) intervention using protocol-based pharmacotherapy management; and (7) others.

The study identified pharmacists through affiliations or verified that the names existed in the pharmacist licence database of the Ministry of Health, Labour and Welfare. Moreover, the number of studies written by pharmacists and non-pharmacists as first authors were compared and further classified according to the seven categories. The affiliations of pharmacists as first authors were classified as follows: hospitals (stratified by bed size), universities, community pharmacies and others.

### Statistical analysis

Categorical variables were compared using Pearson’s chi-squared test or Fisher’s exact test. All statistical analyses were two-tailed. *P* < 0.05 was considered statistically significant.

Interrupted time series analysis (ITSA) was performed to identify the effect of the ICPS certification system for fiscal year 2005 on the number of articles in Japanese. A total of 23 years of annual data points in the pre- and post-implementation periods were available for analysis by linear regression. The model included an intercept (*β*0), baseline trend (*β*1), change in level after the implementation of the certification system (*β*2) and change in trend after the certification system (*β*3) [11].

Statistical analyses were performed using the Statistical Package for the Social Sciences software version 23.0 (IBM Japan).

## Results

### Literature search and selection

**Fig. 1:**
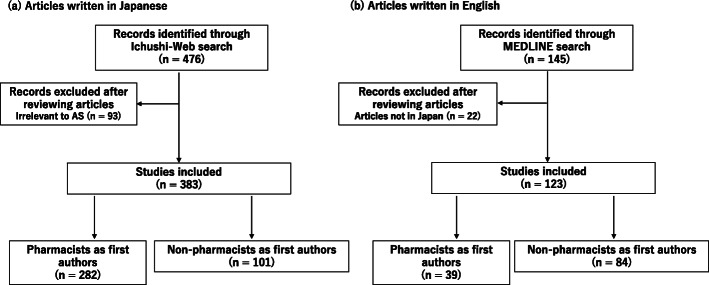
Flow diagrams of the search and selection of literatue relevant to antimicrobial stewardship. The flow indicates the identification of articles written in **a** Japanese on Ichushi-Web and **b** in English on MEDLINE. AS: antimicrobial stewardship

The literature search retrieved 476 and 145 articles from Ichushi-Web and MEDLINE out of which 383 and 123 articles, respectively, were relevant to AS in Japan. Figure 1 depicts the flow diagrams of the selection process. After screening, the study identified 282 (73.6%) and 39 (31.7%) pharmacists as first authors out of 383 and 123 articles in Japanese and English, respectively. The most frequent affiliations of the pharmacists were large-sized hospitals (Table 1).

**Table 1:** Characteristics of affiliations of pharmacists as first authors

	Hospital bed size					
	Small (< 301)	Medium (301–500)	Large (> 500)	University	Pharmacy	Other
Japanese article	49 (17.4)	83 (29.4)	133 (47.2)	12 (4.3)	1 (0.4)	4 (1.4)
English article	1 (2.6)	2 (5.1)	22 (56.4)	13 (33.3)	1 (2.6)	0 (0)

Data were presented as n (%)

### Annual publications of articles in Japanese and English

**Fig. 2:**
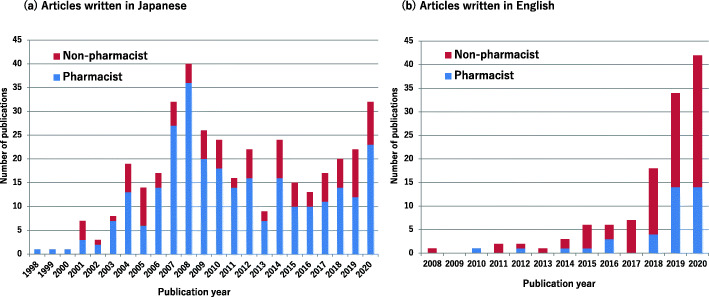
Annual publications of **a** Japanese and **b** English articles in the field of antimicrobial stewardship until 2020. Blue bars indicate pharmacists as first authors; red bars indicates non-pharmacists as first authors

Figure 2 presents a graph of the yearly transition of the publication of articles in Japanese and English. Continued publication was observed for articles written by Japanese pharmacists since 1998, whereas a few articles in English until 2017 were noted.

### Comparing the characteristics of articles written by pharmacists and non-pharmacists

The most frequent content of the articles was intervention relevant to AS for articles written in Japanese and English (Tables 2 and 3). The proportion of articles relevant to AS intervention written by pharmacists, was significantly higher compared with that written by non-pharmacists (Japanese: 56.7% vs. 35.6%; *P* < 0.001; English: 59.0% vs. 39.3%; *P* = 0.041).

**Table 2:** Characteristics of article in Japanese between pharmacist and non-pharmacist first author

	Total (*n* = 383)	Pharmacists as first authors (*n* = 282)	Non-pharmacists as first authors (*n* = 101)	*P*-value
Intervention	196 (51.2)	160 (56.7)	36 (35.6)	< 0.001
TDM	28 (7.3)	25 (8.9)	3 (3.0)	0.051
Surveillance	112 (29.2)	71 (25.2)	41 (40.6)	0.003
Efficacy and adverse event	12 (3.1)	4 (1.4)	8 (7.9)	0.003
Case report	11 (2.9)	6 (2.1)	5 (5.0)	0.167
PBPM	2 (0.5)	2 (0.7)	0	> 0.99
Other	22 (5.7)	14 (5.0)	8 (7.9)	0.273

Data were presented as n (%)

*TDM* therapeutic drug monitoring, *PBPM* protocol-based pharmacotherapy management

**Table 3:** Characteristics of articles in English between pharmacists and non-pharmacists as first authors

	Total (*n* = 123)	Pharmacists as first authors (*n* = 39)	Non-pharmacists as first authors (*n* = 84)	*P*-value
Intervention	56 (45.5)	23 (59.0)	33 (39.3)	0.041
TDM	2 (1.6)	2 (5.1)	0 (0)	0.099
Surveillance	40 (32.5)	8 (20.5)	32 (38.1)	0.053
Efficacy and adverse event	8 (6.5)	2 (5.4)	6 (7.6)	> 0.99
Case report	0	0	0	–
PBPM	0	0	0	–
Other	17 (13.8)	4 (10.3)	13 (15.5)	0.435

Data were presented as n (%)

*TDM* therapeutic drug monitoring, *PBPM* protocol-based pharmacotherapy management

### Effect of ICPS certification system on the publication trend of articles in Japanese written by pharmacists

**Fig. 3:**
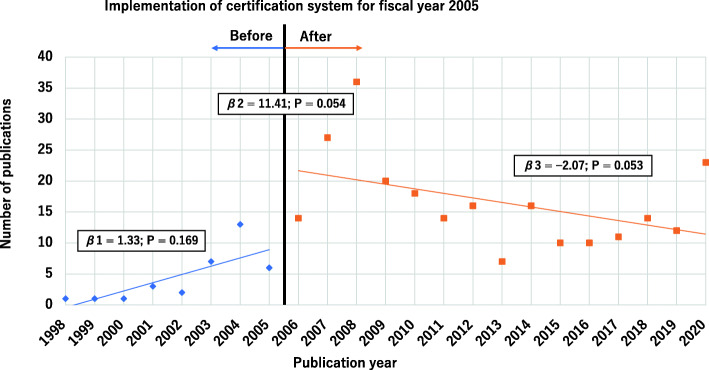
The line graph indicates a segmented linear regression of pharmacists as first authors of articles written in Japanese during the pre- and post-implementation periods. The trend slightly increased before the implementation period [*β*1 = 1.33, 95% confidence interval (CI): −0.62–3.28; *P* = 0.169]. Afterward, the level (*β*2 = 11.41, 95%CI: − 0.23–23.05; *P* = 0.054) increased, whereas the slope decreased (*β*3 = −2.07, 95%CI: −4.16–0.03; *P* = 0.053)

**Fig. 4:**
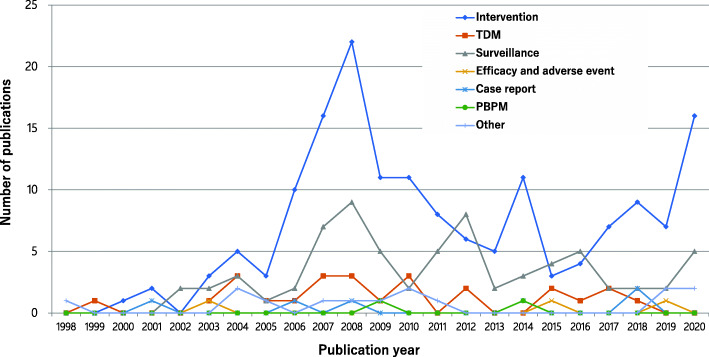
The number of annual publications of pharmacists as first authors of Japanese articles divided into seven categories relevant to antimicrobial stewardship. TDM: therapeutic drug monitoring; PBPM: protocol-based pharmacotherapy management

ITSA indicated a change in publication trend after the implementation of the ICPS certification system. The trend slightly increased before the implementation period [*β*1 = 1.33, 95% confidence interval (CI): − 0.62–3.28; *P* = 0.169]. Afterward, the level (*β*2 = 11.41, 95%CI: − 0.23–23.05; *P* = 0.054) increased, whereas the slope decreased (*β*3 = − 2.07, 95%CI: − 4.16–0.03; *P* = 0.053) (Fig. 3). However, the changes were not statistically significant. Peaks of publications were observed for 2008 and 2020. The study identified several articles relevant to AS intervention for 2008 and 2020 in annual publications (Fig. 4).

## Discussion

To the best of our knowledge, this study is the first bibliometric analysis to report on publications relevant to AS in Japan, which investigates the literature on AS in Japan and presents the scientific contribution of pharmacists.

Continued publications by pharmacists were observed for articles written in Japanese, whereas a growing body of English publications was observed since 2018 (Fig. 2). Moreover, the affiliations of pharmacists as first authors of Japanese and English articles were medium- to large-sized hospitals (Table 1). In the past, various health policies considered effective for AS were enforced. The revised additional reimbursement for infection control in 2010 and 2018 required the employment of pharmacists to promote hospital infection control and AS [7, 12, 13]. The requirements of the additional reimbursement were directed toward the improvement of the size, infrastructure and human resources of large hospitals [10, 14]. The reason underlying this notion is that large-sized hospitals, which includes teaching and university hospitals, foster an environment conducive to AS activities conducted under the evidence-based approach. The study infers that these hospitals lead publications relevant to AS in Japan by publishing their activities. The results of the study can serve as a direction in promoting the evidence-based activities of AS for small- to medium-sized hospitals, which account for approximately 70% of all hospitals in Japan [15].

Several AS guidelines recommended preauthorization and/or prospective audit and feedback interventions by an AS team that comprises physicians and pharmacists specialising in infectious diseases to promote appropriate antibiotic use [6, 16]. An analysis of the characteristics of Japanese articles showed that 160 out of 196 articles (81.6%) about the intervention relevant to AS were written by pharmacists. In addition, the proportion of articles intervention relevant to AS written by pharmacists was significantly higher compared with that written by non-pharmacists (Tables 2 and 3). The characteristics of the articles written by pharmacists indicated that pharmacists largely contributed to AS by providing interventions for appropriate antimicrobial use in hospitals. This result supports the notion that hospital pharmacists with expertise in infectious diseases is an essential element of AS promotion in Japan.

The certification system for specialties in infection control and oncology, which was implemented in fiscal year 2005 was the first system for Japanese pharmacists [4]. Thus, the study estimated the impact of the ICPS certification system on AS activity and research using ITSA. The results revealed that the certification system influenced the increment of publications written by pharmacists (Fig. 3). This result is based on a requirement of at least two peer-reviewed publications relevant to infection control or AS, which influenced the increase in publication. In contrast to the publications in Japanese, the number of articles in English did not increase in the post-implementation period (Fig. 2). The requirement for certification was the publication of research papers in either Japanese or English. Only three English papers have been published in small to medium-sized hospitals (Table 1). It would be challenging to publish English papers in resource-limited settings lacking a mentor to write papers in English.

The study observed peaks in publications at 2008 and 2020 (Fig. 3). The most frequent content of the articles during the peak years was intervention relevant to AS (Fig. 4). The impact model of the certification system and health policies regarding publications could be estimated that considering the term from AS activity to publish an article, and the number of articles reached a peak in 2–3 years later. However, a long-term study is required to examine the effect of the additional reimbursement for AS, which was enforced in 2018, on publications.

The present study has several limitations. First, bibliometric analysis could not be used to evaluate the process and outcome of AS strategies. Moreover, the selected literature included various types of articles, such as notes and letters. Thus, systematic reviews and meta-analyses according to specific topics are required to evaluate the outcomes of the contribution of pharmacists. Second, several affiliation data were duplicated because several of the selected studies were published at the same institutions, particularly large-sized hospitals. These hospitals reported various activities relevant to AS. Finally, the study overlooked the publication trend of articles in English using ITSA due to the small number of articles. A previous study reported that the term ‘antimicrobial stewardship’ in English has become mainstream globally in the past decade [17]. It is possible that the term was simply not mentioned or was denoted by another term (e.g. antibiotic control or management). Moreover, a previous meta-analysis reported that publications on AS in Japan are limited [18]. Thus, continued efforts toward the evidence-based activities for AS strategies as reported pharmacists are required to publish papers in English. In particular, such articles should focus on resource-limited settings, such as small-sized hospitals. Increased commitment by policymakers and stakeholders to support small- to medium-sized hospitals is also further required to develop pharmacists with expertise in AS at the national level [19].

## Conclusion

The study employed bibliometric analysis to determine the contribution of pharmacists by identifying the trends in AS practice. The study observed continued publications written by pharmacists in large-size hospitals and changes in the trend of publications after the implementation of the ICPS certification system. Thus, policymakers and stakeholders should promote and support the evidence-based activities for AS employed by pharmacists in small- to medium-sized hospitals to fill the existing knowledge gap.

## Supplementary Information

**Additional file 1.** Search terms of literature released on Ichushi-Web and MEDLINE.

## Data Availability

The datasets used and analysed in the current study are available from the corresponding author upon reasonable request.

## Abbreviations

AS: Antimicrobial stewardship
ICPS: Infection control pharmacy specialist
ITSA: Interrupted time series analysis
