# Supplementary material for: Bibliometric analysis of pharmacist’s research on antimicrobial stewardship in Japan: an interrupted time series analysis on the implementation of the certification system for infection control pharmacists
Source: J Pharm Health Care Sci. 2021 Nov 1;7:38. doi: 10.1186/s40780-021-00223-w (PMC8559347; doi:10.1186/s40780-021-00223-w)
Supplement: Supplementary file 1 — Additional file 1. Search terms of literature released on Ichushi-Web and MEDLINE. [file 40780_2021_223_MOESM1_ESM.docx]

Additional file 1. Search terms of literature released on Ichushi-Web (A) and MEDLINE (B).

(A) Search terms which used on Ichushi-Web.

((抗感染剤/TH or 抗菌薬/AL) and 適正使用/AL or (抗菌薬適正使用支援/TH or 抗菌薬適正使用支援/AL)) and ((FT=Y OR FTF=Y) LA=日本語 PT=原著論文)

‘抗感染剤’ and ‘抗菌薬’ mean antimicrobial.

‘適正使用’ means appropriate use.

‘抗菌薬適正使用支援’ means antimicrobial stewardship.

‘日本語’ means Japanese (language).

‘原著論文’ means original article.

TH, thesaurus; AL, all fields; FT, loattrfull text; FTF, loattrfree full text; LA, language; PT, publication type

(B) Search terms which used on MEDLINE.

(‘antimicrobial stewardship’[MeSH Terms] OR (‘antimicrobial’[All Fields] AND ‘stewardship’[All Fields]) OR ‘antimicrobial stewardship’[All Fields]) AND (‘japan’[MeSH Terms] OR ‘japan’[All Fields] OR ‘japan s’[All Fields] OR ‘japans’[All Fields])
